# Gut microbiota in perimenopausal atherosclerosis: the estrogen-gut-vascular axis and personalized cardiovascular prevention

**DOI:** 10.3389/fendo.2026.1815352

**Published:** 2026-04-24

**Authors:** Yiying Zhu, Yanhua Li

**Affiliations:** 1 The Second School of Clinical Medicine, Zhejiang Chinese Medical University, Hangzhou, China; 2 Department of General Practice, The Second Affiliated Hospital of Zhejiang Chinese Medical University (Xinhua Hospital of Zhejiang Province), Hangzhou, China

**Keywords:** atherosclerosis, estrogen, gut microbiota, perimenopause, treatment

## Abstract

The risk of atherosclerosis rises markedly in perimenopausal women. The observed discrepancy between the traditional “estrogen cardioprotection hypothesis” and the complex effects of hormone replacement therapy in clinical practice suggests the existence of intermediary mechanisms that are not yet fully understood. Recent research indicates that the gut microbiota may play a pivotal role in this “estrogen paradox”. By integrating current evidence, this review systematically elucidates the core driving function of the “estrogen-gut-vascular axis” in disease progression: declining estrogen levels lead to intestinal barrier dysfunction and associated imbalances in microbial metabolites (e.g. reduced short-chain fatty acids and increased pro-inflammatory metabolites), collectively accelerating atherogenesis. Targeting this axis through dietary modification, microbial therapeutics, and precision hormone interventions may break this pathological cycle. Notably, effective nutritional strategies must consider food matrix, individual microbial metabolic capacity, and timing of intervention. Furthermore, building on extensive research into age-related shifts in gut microbiota, this review proposes the novel concept of ‘gut microbial age’ based on functional metabolic profiles, to quantify the functional state of host–microbiome interactions. This concept aims to provide new perspectives and tools for personalized cardiovascular risk assessment and precise intervention in perimenopausal women.

## 1 Introduction

The topic of women’s health is drawing increasing attention. A particular focus lies in the constellation of symptoms, such as hot flashes, night sweats, urinary incontinence, and emotional lability, that emerge with age, collectively termed perimenopausal syndrome. This phenomenon represents the external manifestation of systemic dysregulation within the female body (1). The primary underlying mechanism is the decline in ovarian function, leading to a progressive reduction in estrogen levels, which subsequently impairs the normal functioning of other endocrine systems and neuroendocrine axes (2). Estrogen, a hormone crucial for cardiovascular protection in premenopausal women, becomes a key factor in reversing the gender disparity in cardiovascular event risk as women enter the perimenopausal period, particularly regarding atherosclerosis (AS) (3). Research indicates that endothelial dysfunction begins during perimenopause and worsens with declining estrogen (4). Specifically, vascular endothelial function is predominantly regulated by nitric oxide (NO) and endothelin-1 (ET-1). Decreasing estrogen levels downregulate NO release and upregulate ET-1 concentration, thereby increasing vascular endothelial permeability and limiting vascular compliance (5, 6). Concurrently, through its influence on metabolic and inflammatory factors, estrogen withdrawal mediates inflammatory responses, promoting plaque formation and destabilization (7, 8), ultimately compromising the entire cardiovascular system.

Multiple studies have found that against the backdrop of estrogen deficiency, the composition, abundance, and function of the gut microbiota undergo significant changes (9). Estrogen and its receptors influence intestinal structure and function through both genomic and non-genomic signaling pathways, indirectly modulating local immunity and inflammatory responses, thereby shaping the gut microbial ecosystem. For instance, studies show that the gut microbiota of postmenopausal women resembles that of men, characterized by a reduction in beneficial taxa such as *Bifidobacterium* and *Ruminococcus*, and a proliferation of potentially harmful bacteria like those within the *Firmicutes phylum* (10).

The gut microbiota has re-emerged as a focal point of research. This immensely populous microbial community inhabiting the digestive tract of humans and other animals maintains a symbiotic relationship with the host. Moving beyond its previously recognized role in digestive health (11), recent years have positioned the gut microbiota and its metabolites as critical diagnostic biomarkers and therapeutic targets (12), revealing its significant influence on other physiological systems (13). Functioning as a virtual metabolic organ, it contributes to intestinal homeostasis while profoundly impacting host physiology, metabolism, and immune function (14). Its metabolites, such as TMAO, can promote the development of atherosclerotic cardiovascular disease by mediating endothelial dysfunction, inflammation, and lipid metabolism (15).

Recent research has intensified exploration of the estrogen-gut axis. Investigating the association between estrogen and the gut microbiota offers promising avenues for improving women’s health through microbial modulation (16). For example, studies in ovariectomized (OVX) mouse models have identified an abundance of Alistipes inops that accelerates tryptophan degradation, inducing depression-like behavior, while also acting as a key mediator for melatonin in ameliorating mood disorders (17).

This review proposes the gut-vascular axis as a critical bridge connecting hormonal withdrawal and vascular injury. We aim to elucidate how, in the context of declining estrogen, this axis modulates and influences vascular structure and function during the atherosclerotic process. We will focus on delineating the interrelationships between estrogen levels, the gut microbiota, and AS. Specifically, we will explore the association and mechanistic role of the gut in AS development in this key demographic, discuss the implications of gut microbiota modulation for vascular function, and evaluate potential therapeutic strategies.

## 2 Structural transformation of the gut microbiota during perimenopause

The withdrawal of estrogen in perimenopause directly eliminates the host’s sex-specific microenvironment, which is first reflected in a fundamental restructuring of the overall architecture and diversity of the gut microbiota. The diversity of this ecosystem can be assessed through two key dimensions: α-diversity, which measures species richness and evenness within a single sample, with higher α-diversity generally associated with microbiome stability and host health; and β-diversity, which quantifies compositional differences between samples and can reveal the impact of environment, host status, or external interventions on microbial structure (18). Cross-sectional and prospective studies consistently show that the gut microbial α-diversity in postmenopausal women is significantly lower than in their premenopausal counterparts and progressively converges with the male profile (19, 20). This attenuation of diversity has been associated with decreased microbiome stability and adverse metabolic health markers in observational studies (21).

The compositional shift is a dynamic balance: a decline in the abundance of *Firmicutes phylum* members, particularly key butyrate-producing genera such as *Roseburia* and *Faecalibacterium*, which are positively associated with health, and a relative increase in the proportion of the potentially pro-inflammatory *Bacteroidetes phylum* (22). Concurrently, there is an abnormal proliferation of genera significantly linked to host lipid metabolism disorders, such as *Lactobacillus* and *Ruminococcus* (23). This structural imbalance marks the transition of the gut microecology from a “stable symbiosis” to a state of “dysbiosis.”

Both clinical studies and animal experiments (typically using age-based or ovariectomy models) consistently demonstrate significant gut microbiota remodeling during the critical physiological transition of perimenopause (**Table 1**). The core features include reduced microbial diversity, imbalanced community structure, and an increased relative abundance of pro-inflammatory taxa. At the functional level, the metabolic output of the microbiota shifts towards “less beneficial, more harmful” activities, exemplified by a decrease in protective metabolites like short-chain fatty acids and an enhancement of potentially detrimental metabolic pathways. Furthermore, estrogen has been confirmed to regulate systems beyond reproduction by modulating the abundance of specific microorganisms (**Table 2**). For instance, in lipid metabolism, estrogen deficiency leads to an increase in signature microbes (e.g. *Lactobacillus* and *Ruminococcus*) that are significantly negatively correlated with acylcarnitine synthesis. Estrogen supplementation can partially restore microbial community structure, thereby alleviating lipid disorders (24).

**Table 1:** Changes in the composition and metabolic function of the gut microbiota during the perimenopause.

Study type	Author	Sample	Methods	Microbiota composition findings	Metabolic functional findings	Implications
Clinical	Santos-Marcos et al. (86)	Premenopausal (n=17) vs. Postmenopausal (n=20) women; Men (n=39) as controls	16S rRNA sequencing	1 .↓ *Firmicutes、Lachnospira* and *Roseburia* 2. ↑ *Prevotella、Parabacteroides、Bilophila* 3.No difference in α-diversity Difference in β-diversity	1. ↓ GLP-1 plasma levels、propanoate/butanoate metabolism 2. ↑ IL-6 and MCP-1 plasma levels	Suggests a shift towards a pro-inflammatory microbiota profile post-menopause.
Clinical	Peters et al. (95)	Premenopausal (n=295) vs. Postmenopausal (n=1,027) women; Men (n=978) as controls	Shotgun metagenomics	1. ↓ Shannon diversity index:α-diversity PERMANOVA:β-diversity(R² = 0.13%, P = 0.009) 2. ↓ Escherichia coli-Shigella spp., Oscillibacter sp. strain KLE1745, Akkermansia muciniphila, Clostridium lactatifermentans, Escherichia coli, Parabacteroides johnsonii, and Veillonella seminalis 3. ↑ Bacteroides sp. strain Ga6A1, Prevotella marshii, Sutterella wadsworthensis	1. ↑ Sulfate transport system 2. ↓ Bacterial β-glucuronidase	Highlights diversity loss and specific functional pathway changes.
Animal	Dai et al. (96)	Young vs. Middle-aged female rats	16S rRNA + LC-MS	1. ↑ Clostridia, Anaerovorax, Rikenella, Tyzzerella_3, Carnobacteriaceae, Atopostipes 2. ↓ Bacteroides, Lactobacillus, Erysipelatoclostridium, Anaeroplasma, Anaerofustis, Parasutterella, and Enterococcus	**↑** Gut hormones (Ghrelin, GLP-1, NPY)	Provides mechanistic insight into gut-brain axis changes with age.
Animal	Wei et al. (85)	OVX rats vs. Control rats	16S rRNA + UHPLC-MS/MS	1. PCoA:significant difference in β-diversity 2. ↑ Ruminococcus、Intestinimonas 3. ↓ abundance of Aggregatibacter segnis, Bifidobacterium animalis, and Acinetobacter guillouiae 4. P>0.05: Firmicutes	1. ↑ ABC transporter pathway 2. ↑ Purine metabolism pathway	Links microbiota shifts to specific host metabolic alterations in a model system.

NMDS, Nonmetric multidimensional scaling analysis; PCoA, Principal coordinate analysis; OVX, Ovariectomized Model.

P>0.05: NO significant difference.

↑: an increase or enhancement in the microbiata composition and metabolic functions or mechanisms.

↓: a decrease or weakening in the microbiata composition and metabolic functions or mechanisms.

**Table 2:** Changes in trends and mechanisms of action of different microbiota during the perimenopause.

Taxonomic level	Microbiota name	Postmenopausal change trend	Mechanism of action
Phylum	Bacteroidetes	↑ (in some taxa)	1. Sulfate competition and β-glucuronidase activity affect hormone metabolism. 2. Pro-inflammatory potential (LPS) exacerbating low-grade inflammation and altering energy metabolism.
Phylum	Firmicutes	↓ (specific beneficial genera)	1. Reduction of protective genera (e.g. Roseburia) leading to insufficient SCFA synthesis.
			2. Consequences: barrier dysfunction, bone metabolism imbalance, and glucose/lipid metabolism dysregulation.
Genus	Prevotella	↑	The expression of pro-inflammatory molecules (LPS) activates immune pathways (TLR2/4) and drives low-grade inflammation.
Genus	Ruminococcus	↓	Decreased fiber-degrading enzyme activity reduces SCFA production. This weakens its anti-inflammatory and metabolic regulatory roles, thereby exacerbating metabolic disorder.
Genus	Akkermansia	↓	1. Affects hormone deconjugation reactions.
			2. Disrupts mucus layer homeostasis, reducing hormone reactivation and compromising barrier function.
Genus	Dorea	↑ (requires further validation)	Hydrogen gas production influences redox state and metabolism. Its increase may reflect, rather than cause, a dysbiotic structure linked to metabolic disorders; however, causality remains unclear.
Genus	Sutterella	↑	Associated with mucosal inflammation, produces specific metabolites affecting epithelial integrity; its enrichment correlates with increased blood pressure and cardiovascular risk.
Genus	Parabacteroides	↓	Participates in bile acid transformation, affecting the FXR signaling pathway; its decline is a marker of community shift linked to lipid metabolism dysregulation.
Genus	Oscillibacter	↓	Associated with α-hemolysin/cyclohemolysin transport systems, influencing blood lipid homeostasis.
Genus	Veillonella	↓	Decline in lactate utilization and propionate generation; its reduced abundance is associated with postmenopausal microbiota remodeling and may affect metabolic homeostasis.
Species	Escherichia coli-Shigella spp.	↓ (in healthy individuals)	In healthy individuals, enhanced immune clearance is beneficial. In disease states, virulence factors promote inflammation. This contrasting pattern highlights differences that depend on health status.
		↑ (in breast cancer)	

Data frome Shuting Yu et al. (97)、Leena Sapra et al. (98)、Jiaqing Ji et al. (99).

↑: an increase in the abundance of the microbiota.

↓: a decrease in abundance of the microbiota.

In summary, despite complexities in research models and intervention outcomes, a substantial body of evidence from both clinical and preclinical studies supports the concept that the destabilization of the gut microbial “niche”, accompanying perimenopause constitutes a key pathological link between estrogen withdrawal and systemic metabolic and inflammatory disturbances, thereby driving increased cardiovascular risk.

## 3 The gut microecological mechanisms of AS in perimenopause

Perimenopausal women are at a significantly elevated risk of AS, a process closely linked to systemic metabolic-immune network disturbances triggered by the withdrawal of estrogen levels. The gut microecology, as a key component of this network, interacts intricately with the neuroendocrine and immunometabolic systems, collectively forming the pathogenic basis driving AS development. The core mechanism involves a disruption in the dynamic equilibrium between intestinal barrier integrity, protective microbial metabolites, and harmful bacterial products.

Estrogen acts as a central regulator in maintaining the structural and functional integrity of the intestinal barrier. Estrogen receptors are widely expressed in intestinal epithelial cells and immune cells, and their activation status critically shapes the local microenvironment (25). Basic research confirms that estrogen, primarily via the ERβ receptor, can directly upregulate the expression of tight junction proteins (e.g. Claudins, Occludin, ZO-1) and activate signaling pathways such as EGFR/PI3K-Akt to strengthen the epithelial barrier (26, 27). Concurrently, estrogen helps maintain the abundance of key mucus-degrading bacteria like *Akkermansia muciniphila* (28, 29). Preclinical studies demonstrate that supplementing with *Akkermansia muciniphila* can repair the barrier by significantly upregulating ZO-1 and Occludin expression, markedly reduce circulating lipopolysaccharide (LPS) levels, and inhibit arterial plaque inflammation. These effects are independent of lipid modulation (30). However, in the context of perimenopausal estrogen withdrawal, these defensive mechanisms are directly impaired, leading to a significant increase in intestinal permeability, known as “leaky gut”. For instance, clinical studies have confirmed increased intestinal permeability in perimenopausal women, accompanied by elevated markers of microbial translocation (31). LPS entering the bloodstream acts as a critical pathogenic messenger. By binding to Toll-like receptor 4 (TLR4) on immune cells like macrophages, it activates the classic MyD88/NF-κB pro-inflammatory pathway, driving M1 macrophage polarization and a cytokine storm, directly exacerbating plaque inflammation and instability (32, 33). This collapse of barrier function is thought to open a gateway for gut microbes and their metabolites to enter systemic circulation, thereby contributing to the pathological foundation for systemic inflammation and immune activation (34).

Regarding protective metabolites, short-chain fatty acids (SCFAs, such as butyrate, acetate, and propionate) and bile acids (BAs) play vital roles. SCFAs are produced by specific gut bacteria (e.g. *Clostridium clusters*) fermenting dietary fiber. Studies indicate a positive correlation between estrogen levels and Clostridium abundance, with the latter efficiently synthesizing butyrate and acetate, which are linked to reduced estrogen-related disease risks (35, 36). Locally, butyrate promotes regulatory T cells (Tregs) differentiation and anti-inflammatory cytokine secretion (37, 38); propionate exerts systemic anti-inflammatory and organ-protective effects via Tregs (39); and acetate, at the vascular level, can inhibit the proliferation of pathogenic bacteria like *Lactococcus lactis* within plaques (40). All three SCFAs can inhibit ox-LDL-induced cellular inflammatory damage by blocking the NLRP3/Caspase-1 pathway (41). Estrogen decline impedes these protective mechanisms by reducing SCFAs levels. For example, animal studies found that OVX mice had lower SCFAs concentrations and more severe glucolipid metabolic disorders than controls—a phenotype reversible via microbiota modulation (42). The liver-gut signaling axis mediated by the farnesoid X receptor (FXR) is crucial for maintaining cholesterol homeostasis. Its proper function enhances cholesterol excretion and inhibits excessive hepatic bile acid synthesis, thereby lowering circulating cholesterol levels (43). Estrogen participates in regulating bile acid synthesis and enterohepatic circulation. Its decline weakens the FXR pathway-mediated reverse cholesterol transport capacity, exacerbating lipid accumulation (44). BAs themselves can inhibit platelet activation via TGR5 receptor signaling, exerting antithrombotic effects (45). In mice, OVX impairs fecal bile acid secretion, which is restored upon estradiol supplementation (35).

Parallel to the attenuation of protective signals is the amplification of damaging ones, with the TMAO pathway being particularly critical (46). Gut microbiota metabolize dietary choline and other precursors into trimethylamine (TMA), which is subsequently oxidized in the liver to TMAO. Clinical observations reveal that fasting plasma TMAO concentrations are significantly higher in postmenopausal women compared to age-matched men and premenopausal women (47). In perimenopausal models, estrogen withdrawal-induced dysbiosis can drive TMAO elevation (48). Circulating TMAO levels are widely recognized as an independent risk biomarker and potential pathogenic mediator for cardiovascular disease (49), and is proposed to accelerate AS through multiple mechanisms. At the cellular level, it activates macrophage MAPK/JNK signaling to promote foam cell formation (50); exacerbates inflammation and proliferation in vascular smooth muscle cells via the JNK-NF-κB pathway, impairing plaque stability (51, 52); and can abnormally elevate Beclin1 levels, interfering with autophagy in vascular endothelial cells (53). Systemically, TMAO can regulate relevant miRNAs, disrupt metabolic rhythms, alter pro-atherosclerotic protein expression profiles (54, 55), and amplify risk by interfering with the bile acid FXR pathway and enhancing platelet activity (56, 57).

Notably, “leaky gut” is not merely a background condition but a key factor enabling the direct toxic effects of precursors like TMA. TMA, the precursor to TMAO, may act as an independent toxic mediator upon entering circulation. Emerging research in aging rat models suggests that TMA can directly damage vascular smooth muscle cells (58), broadening the TMAO-centric pathogenic framework. However, its relative contribution and clinical significance in human perimenopausal AS require further exploration.

The gut microbiota is a key regulator of endogenous estrogen metabolism. After estrogen is inactivated and conjugated with glucuronic acid in the liver, it is excreted via bile into the intestine. Reactivation to its bioactive free form relies on bacterial enzymes, primarily β-glucuronidase (59). Gut bacteria rich in this enzyme, such as *Levilactobacillus brevis* and *Lacticaseibacillus rhamnosus*, hydrolyze conjugated estrogen, allowing its reabsorption into the bloodstream via enterohepatic circulation (60). Higher β-glucuronidase activity in premenopausal women signifies that their gut microbiota serves as a crucial “peripheral hub” for maintaining endogenous estrogen homeostasis (61, 62). Consequently, the composition and function of the gut microbiota directly influence the bioavailability of systemic estrogen, thereby regulating a range of physiological processes from bone metabolism to cardiovascular health (63, 64). This discovery provides a strong theoretical basis for interventions using probiotics or prebiotics to manage perimenopausal symptoms.

**Figure 1:**
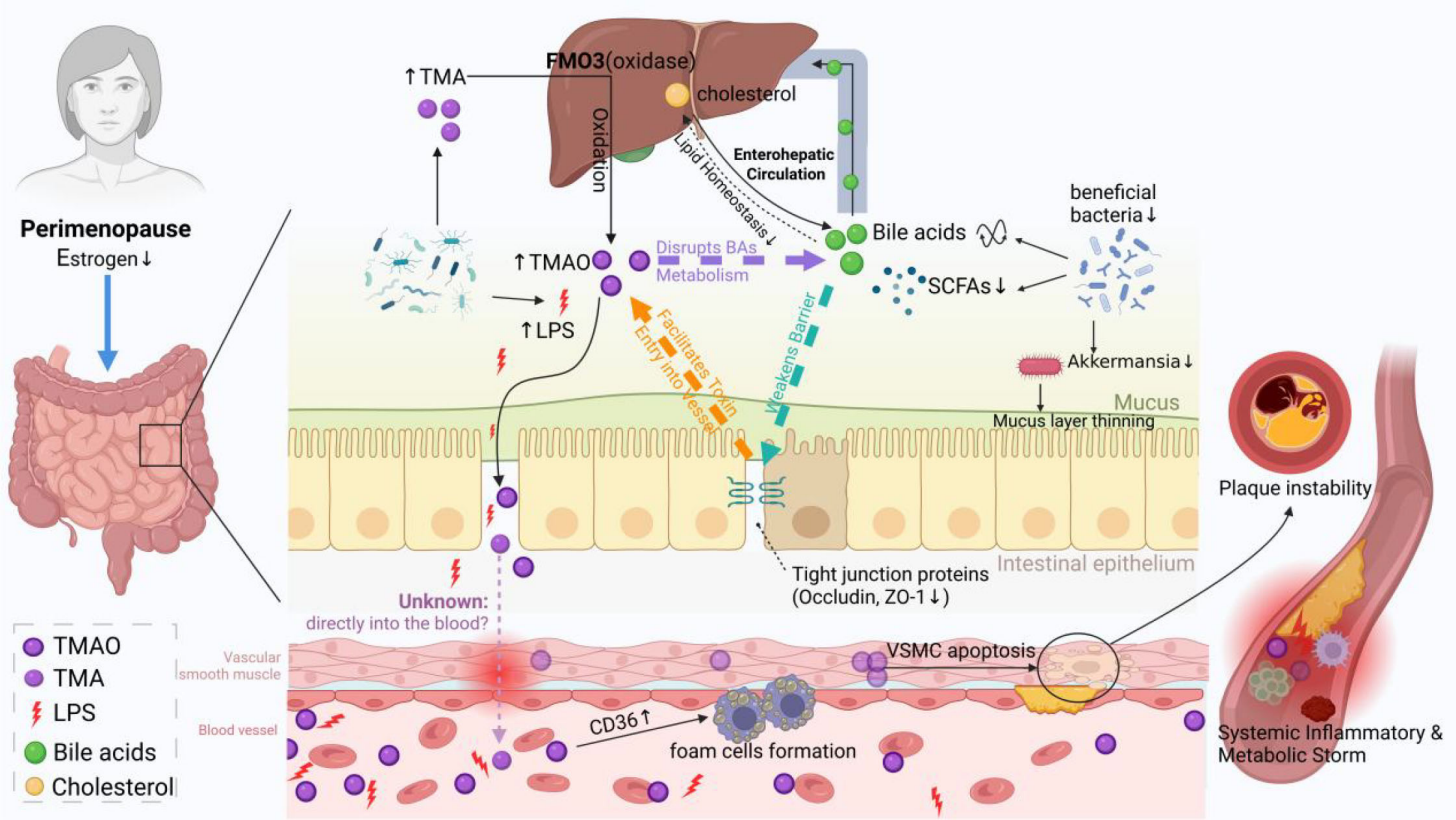
Estrogen deficiency drives a vicious cycle of gut-vascular axis. Orange Axis: The decline in estrogen levels directly downregulates the expression of tight junction proteins (Occludin, ZO-1) in intestinal epithelial cells, impairing intestinal barrier integrity and leading to “leaky gut”. This structural dysfunction allows gut-derived endotoxins (LPS) and microbial metabolites (TMAO) to continuously translocate into systemic circulation, thereby activating systemic immune-inflammatory responses. Green Axis: Concurrently, reduced production of protective metabolites by the gut microbiota—particularly short-chain fatty acids (SCFAs)—and disturbances in bile acids (BAs) signaling pathways further exacerbate intestinal barrier dysfunction, amplifying the “leaky gut”. Purple Axis: Elevated TMAO levels interfere with liver metabolic homeostasis by modulating bile acid composition, thereby aggravating systemic lipid metabolism disorders. Once initiated, these three pathological axes form a self-reinforcing vicious cycle. The resulting harmful metabolites can directly act on vascular smooth muscle and endothelial cells, promote foam cell formation, trigger inflammatory responses, and ultimately destabilize atherosclerotic plaques, evolving into an uncontrollable systemic inflammatory and metabolic storm. Created in https://BioRender.com.

In the early stages of disease, ‘leaky gut’ coupled with the attenuation of protective signals may jointly initiate systemic metabolic-immune network disturbances. As the disease progresses, amplifying damage mechanisms like TMA/TMAO pathway activation may gradually dominate, forming a self-reinforcing vicious cycle (**Figure 1**). However, identifying the specific dominant axis and the spatiotemporal evolution of these processes remains a key focus for future research. Interventions aimed at reinforcing the barrier, restoring protective signals, and blocking damaging products represent a novel precision medicine paradigm for preventing and treating cardiovascular disease in perimenopausal women.

## 4 Gut microbiota-based prevention and therapeutic strategies for AS in perimenopause: translational prospects

The aforementioned research elucidates the potential mechanisms underlying perimenopausal cardiovascular risk from various dimensions, including microbial structure, or specific metabolites (TMAO, acylcarnitines). The OVX model, which simulates estrogen withdrawal, can induce specific gut dysbiosis, subsequently driving systemic lipid metabolism disorders and ultimately accelerating AS. Conversely, interventions targeting this axis—either through estrogen supplementation or direct transplantation of a healthy microbiota—can effectively reverse these pathological processes (65). Collectively, these findings establish the “gut microbiota” as an indispensable and central pathological mediator linking “estrogen withdrawal” to “atherosclerosis”. This provides a solid foundational rationale for all prevention and treatment strategies targeting the gut microecology.

### 4.1 Dietary intervention

Dietary intervention serves as a foundational strategy for shaping the gut microbiota and, consequently, preventing and treating AS in perimenopause (66). Its core lies in utilizing specific food components to precisely modulate gut microecological function, thereby yielding systemic cardiovascular and metabolic benefits. Taking soy and its products—rich in plant protein and isoflavones—as an example, the longstanding controversy over their cardiovascular effects has been clarified by high-level evidence: a meta-analysis of 46 RCTs indicated that isolated soy isoflavones primarily improve total cholesterol (TC) and apolipoprotein B (ApoB), whereas intact soy protein containing isoflavones more comprehensively optimizes triglyceride (TG), TC, low-density lipoprotein cholesterol (LDL-C), and ApoB levels, thereby improving the lipid profile in postmenopausal women (67). However, efficacy exhibits significant individual heterogeneity, which appears to be fundamentally influenced by the host gut microbiota’s ability to convert daidzein into the highly active metabolite equol (68). Similarly, foods such as pinto beans (PB) exhibit dual “prebiotic-phytoestrogen” properties: their dietary fiber promotes the proliferation of beneficial bacteria like Bacteroides and SCFA production to reinforce the intestinal barrier; their phytoestrogen components and enhanced β-glucuronidase activity jointly promote the enterohepatic circulation of endogenous estrogen, exerting potential protective effects through both metabolic and endocrine pathways (69). Furthermore, quercetin, a natural flavonoid in fruits and vegetables, prevents AS in postmenopause by activating the KEAP1/NRF2 endogenous antioxidant pathway, thereby inhibiting ferroptosis in vascular endothelial cells (70). Collectively, this evidence indicates that effective nutritional recommendations must comprehensively consider the specific form of food, individual microbial metabolic capacity, and the timing of intervention.

### 4.2 Microbial preparations

The direct supplementation of probiotics or the design of synbiotics aims to rapidly correct menopause-associated dysbiosis. The core concept has shifted from altering microbial structure to conferring key metabolic functions. Specific probiotic strains (e.g. *Lactobacillus plantarum ATCC 8014*) have been shown to ameliorate bile acid metabolic abnormalities induced by a high-fat diet in OVX mice by upregulating genera such as *Allobaculum* and *Olsenella* in the ileum, which may promote cholesterol conversion and excretion, contributing to lipid-lowering benefits in OVX mice (71). A more promising strategy involves the synergy between probiotics and phytochemicals. Probiotics can independently improve endothelial function (72), and their combination with phytoestrogens (e.g. red clover isoflavones) can produce a “1 + 1>2” effect: probiotics enhance the bioavailability and conversion efficiency of isoflavones, thereby amplifying their estrogen-like activity and clinical efficacy in alleviating vasomotor symptoms in perimenopausal women (73, 74). For perimenopausal women with contraindications to hormone therapy, synbiotic formulations composed of prebiotics and specific probiotics offer a safer alternative or adjunctive strategy. Animal studies suggest that a synbiotic containing β-glucan prebiotics and specific strains (e.g. *Lactobacillus fermentum JS*) can effectively reverse OVX-induced gut dysbiosis (e.g. normalizing the aberrant Firmicutes/Bacteroidetes ratio), showing efficacy comparable to estrogen-positive controls in these models. More importantly, this preparation systemically improves insulin resistance and abnormal lipid profiles while avoiding the uterine hyperplasia risk associated with traditional estrogen therapy, achieving a balance between efficacy and safety (75). Representative traditional Chinese medicine (TCM) compounds, such as Liuwei Dihuang Wan and Taohong Siwu Tang, provide examples of systemic regulation. Through multi-component synergy, they modulate the TMA-TMAO metabolic axis (48)or improve the “microbiota-platelet function/amino acid metabolism” network (76), holistically correcting the metabolic-inflammatory disturbances associated with the perimenopausal syndrome of “blood deficiency and blood stasis.”

### 4.3 Estrogen replacement therapy

Traditionally, supplementation with exogenous estrogen has been viewed as a direct means to correct metabolic disturbances. However, recent studies, primarily from preclinical models, suggest that its systemic protective effects may be mediated by or are highly dependent on an intact and functionally competent gut microbiota, making microbial status a key determinant of inter-individual therapeutic efficacy. In OVX mouse models, the ability of estrogen (e.g. estradiol benzoate) to improve hepatic lipid metabolism and reduce adiposity depends on its capacity to remodel gut microbiota structure and drive the production of specific protective metabolites—acylcarnitines (24). This supports the role of the gut microbiota as a necessary biological intermediary for downstream estrogen signaling. Further studies found that estrogen and its plant analogs can significantly upregulate the expression and activity of intestinal alkaline phosphatase (IAP) secreted by enterocytes, thereby performing the dual critical functions of reducing LPS generation and improving microbial structure (29). This suggests that IAP, as a mediator of estrogen action, may be an important intervention target for ameliorating AS in perimenopause. Hormone therapy (HT) can partially reverse menopause-associated duodenal dysbiosis, inducing a “rejuvenating” shift toward a premenopausal state (77).

However, the existence of the “estrogen paradox” highlights the complexity of perimenopausal intervention. In individuals with pre-existing severe baseline dysbiosis, exogenous estrogen may fail to comprehensively correct microbial imbalances (e.g. the *Firmicutes/Bacteroidetes* ratio) and might instead differentially affect specific genera (e.g. increasing *Prevotella*) or even worsen some metabolic parameters (e.g. further reducing butyrate levels) (78).

## 5 Research gaps and future research directions

### 5.1 Gut microbial age

Multiple studies have reported age-related changes in the gut microbiota, not only identifying midlife as a critical turning point in microbial community evolution, but also emphasizing the importance of analyzing host-microbiome interactions from a metabolic functional perspective (79, 80). Building on this, studies have developed biological age prediction models such as gAge (81) and Viome (82). Compared to traditional biological age indicators reliant on blood biochemical parameters, these models demonstrate reliable predictive value (83). Accordingly, this article proposes the concept of ‘gut microbial age’. This refers to an integrative biological metric, constructed via machine learning models based on the functional signature profile of the host’s gut microbiome (e.g. protein families (Pfams), Kyoto Encyclopedia of Genes and Genomes (KEGG) pathways), designed to quantitatively reflect the host’s physiological aging state (80). Its core premise is that this metric does not rely on microbial taxonomic composition but instead focuses on the functional evolution of the microbial community, thereby characterizing the functional aging state of the gut microbiome as the host ages. The perimenopausal period, a unique stage in a woman’s lifespan where declining estrogen levels may disrupt the pre-existing stable microbial state, makes identifying superior monitoring indicators crucial. A recent systematic review integrating evidence from humans and various animal models (84), utilizing multi-omics approaches like metagenomics, metatranscriptomics, and metabolomics, further underscores the sexual dimorphism of the gut microbiota and highlights the significant role of sex hormones in regulating its diversity and abundance. Therefore, ‘gut microbial age’ holds potential as a biomarker to reveal the dynamic association between host-microbiome interactions during aging and to mark key physiological transition phases. It offers promise as a novel, non-invasive clinical indicator for assessing an individual’s physiological aging rate and cardiovascular disease risk, although its sensitivity and specificity require validation in large-scale studies.

### 5.2 Limitations

However, given that most existing studies are cross-sectional, establishing causality and temporality between perimenopause and changes in ‘gut microbial age’ remains challenging. Future research needs to investigate whether ‘gut microbial age’ can sensitively capture this perimenopause-associated dysbiosis, thereby providing more context-specific insights than generalized age-prediction models and reflecting early AS risk ahead of traditional clinical markers. Longitudinal cohort studies in perimenopausal women, dynamically monitoring changes in gut microbiome functional features from the menopausal transition to early postmenopause, alongside interventional studies using animal models (e.g. OVX models) to verify the direct regulatory effect of estrogen changes on ‘gut microbial age’ are warranted.

It is also important to note the limitations inherent in the models and experimental systems employed. The heavy reliance on preclinical models, particularly the OVX model, constitutes a core constraint. First, surgical ovariectomy induces an abrupt and complete deprivation of estrogen, which stands in sharp contrast to the prolonged and fluctuating decline in hormone levels characteristic of the natural perimenopausal transition in women. Second, interspecies differences in microbiota composition exist; for instance, the OVX model fails to fully recapitulate the Firmicutes-related shifts observed in humans (84, 85), highlighting the inadequacy of conventional animal models in mimicking the complexity of the human neuro-immune-endocrine network. In contrast, humanized or genetically engineered models that better simulate human physiological complexity (86), along with emerging organ-on-a-chip technologies—such as gut-on-a-chip microfluidic platforms (GMoC)—demonstrate considerable potential to address these limitations (87). These platforms can finely reproduce the physicochemical gradients of the human intestinal microenvironment, offering promising avenues for elucidating estrogen-microbiota interactions under more physiologically relevant conditions. Thus, while OVX models provide robust evidence supporting the causal role of estrogen in shaping the gut microbiota and metabolic phenotypes, translating these findings to human physiology requires caution. Ultimately, determining the precise timing, key microbial groups involved, and their functional consequences in humans will depend on well-designed prospective longitudinal cohort studies for validation.

The factors influencing the gut microbiota are complex. Microbiota variation may be more strongly influenced by other factors such as diet, host genetics, and early microbial exposure. A study of multi-site microbiota in 242 healthy humans found that the metagenomic carriage of metabolic pathways remained stable between individuals, with ethnicity/race identified as one of the strongest associated factors, while associations with other phenotypes (e.g. BMI, sex) were generally weaker (18). This contrasts with later research emphasizing sex differences (88), suggesting that when constructing ‘gut microbial age’ models for perimenopausal women, strong confounding factors like race and diet may obscure the subtle functional change signals driven by hormonal fluctuations. Consequently, future studies must rigorously control for these confounders to specifically identify core functional features related to perimenopausal status and validate model generalizability across diverse population cohorts.

Additionally, population heterogeneity presents a major challenge in translational research. For example, the “equol-producer phenotype” is not only a bottleneck in translating findings from animal models to humans but also a key factor confounding data in human studies (89).

Finally, at the therapeutic intervention level, probiotic interventions in postmenopausal women appear to alter the metabolic functions of the microbiome more than its overall taxonomic structure (90). This supports a “function-empowering” intervention strategy, which aligns well with the concept of ‘gut microbial age’ as a functional metric. The “estrogen paradox” observed in individuals with pre-existing severe dysbiosis (78) implies that the controversy surrounding the cardiovascular effects of menopausal hormone therapy (MHT) may partly stem from individual differences in pre-existing ‘gut microbial age’ or state. The previously mentioned therapeutic window of opportunity within the first 5 years postmenopause (91) further suggests that future MHT application requires personalized assessment and intervention. Furthermore, it must be noted that much of the current clinical evidence supporting the aforementioned intervention strategies, including studies on probiotics or MHT, often stems from research with limited sample sizes. Such small sample sizes constrain the statistical power of these studies, potentially increasing the risk of false-positive or false-negative findings, and may compromise the precision of effect size estimates as well as the generalizability of the conclusions. Therefore, future work requires well-designed, sufficiently powered large-scale randomized controlled trials to verify these preliminary findings and to provide a solid basis for personalized interventions.

In summary, future research should focus on establishing multi-omics longitudinal cohorts in perimenopausal women. Analytically, priority should be given to developing explainable artificial intelligence models. Their core value lies not merely in improving prediction accuracy at the population level but in parsing the sources of individual variation and enabling fine-grained stratification of populations based on gut microbiota profiles, functional signatures, and clinical phenotypes, thereby providing a computational framework for subsequent personalized interventions. Study design must be based on deeply phenotyped cohorts that systematically collect key data reflecting individual differences (e.g. estrogen metabolism capacity, baseline microbiota ecotype) beyond conventional metrics.

### 5.3 Future research directions

During the validation phase, it is essential not only to test the overall model performance using independent cohorts but also to specifically evaluate its predictive stability and generalizability within critical subgroups (e.g. different menopausal stages, different microbiota states). Integrating high-throughput sequencing technologies like metagenomics (92) is crucial for providing deeper microbial characterization and functional analysis, which is vital for predicting ‘gut microbial age’. Model interpretability analyses should be employed to quantitatively dissect the impact of different microbial features on disease risk.

Specifically, research needs to: 1) Identify which microbial species or functional pathways driving the acceleration of ‘gut microbial age’ during the specific perimenopausal stage are directly linked to atherosclerotic plaque formation; 2) Elucidate how perimenopausal hormonal changes influence the trajectory of ‘gut microbial age’; 3) Explore whether modulating these targets through diet, probiotics, or microbiota transplantation can simultaneously delay both gut microbial aging and AS progression; 4) Based on these mechanistic insights and individual microbiota characteristics, design and validate intervention strategies, such as dietary modifications, prebiotics, or microbiota transplantation, targeting specific functional pathways within a defined ‘window of opportunity’ (e.g. within 5 years postmenopause).

Although ‘gut microbial age’ is conceptually promising, it must be clearly recognized that transitioning from current associative evidence to a clinically reliable biomarker requires overcoming multiple barriers outlined above, including data heterogeneity, model generalizability, causal validation, and individual variation. Future research designs should not stop at optimizing ‘gut microbial age’ as a predictive tool but should treat it as a dynamic, integrative systems biology indicator, thereby truly enabling the leap from population-level epidemiological associations to individualized prevention and treatment.

## 6 Conclusions

This review systematically elucidates the core role of the “estrogen–gut–vasculature axis” in the pathogenesis and progression of AS during the perimenopausal period. Evidence suggests that the decline in estrogen levels, coupled with subsequent gut microbiota dysbiosis, may collectively drive vascular inflammation and plaque advancement, thereby forming the pathological basis for accelerated AS at this stage (93). Notably, although the trajectories of sex hormone fluctuations and microbiota sexual dimorphism are highly synchronized, direct evidence linking hormones to the microbiota in women is lacking. This reveals the existence of a more complex regulatory network, likely mediated by multiple factors such as immunity and metabolism (94). Within this context, ‘gut microbial age’ as an integrative metric based on functional metabolic profiles, not only offers a novel perspective for risk prediction but also holds future promise. By integrating individual microbiota profiles, menopausal stage, and intervention timing, it could be used to identify subgroups likely to benefit from MHT and to design personalized microbiota-targeted interventions (e.g. prebiotics, dietary modifications). However, the clinical application of this central axis and ‘gut microbial age’ requires further validation through cross-racial, multi-center longitudinal cohorts and mechanistic studies.
